# Rational application of EGFR-TKI adjuvant therapy in patients with completely resected stage IB-IIIA EGFR-mutant NSCLC: a systematic review and meta-analysis of 11 randomized controlled trials

**DOI:** 10.1186/s12885-023-11194-6

**Published:** 2023-08-01

**Authors:** Shu-Ling Zhang, Xiao-Fang Yi, Le-Tian Huang, Li Sun, Jie-Tao Ma, Cheng-Bo Han

**Affiliations:** Department of Oncology, Shengjing Hospital of China Medical University, Shenyang, 110004 China

**Keywords:** Adjuvant therapy, Epidermal growth factor receptor tyrosine kinase inhibitor, Meta-analysis, Non-small-cell lung cancer, Mutation

## Abstract

**Purpose:**

To determine the role and rational application of epidermal growth factor receptor tyrosine kinase inhibitor (EGFR-TKI) adjuvant therapy in patients with completely resected stage IB-IIIA EGFR-mutant non-small-cell lung cancer (NSCLC).

**Method:**

Randomized controlled trials (RCTs) that compared the survival outcomes between adjuvant EGFR-TKIs and adjuvant chemotherapy or a placebo, or between different EGFR-TKI treatment durations for resected NSCLC, were eligible for inclusion. Disease-free survival (DFS) and overall survival (OS) with hazard ratios (HRs) and 95% confidence intervals (CIs) were calculated as effective measures using random-effect or fixed-effect models. Subgroup analysis was also performed.

**Results:**

Eleven RCTs involving 2102 EGFR-mutant NSCLC patients with or without EGFR-TKI adjuvant therapy were included. For all stage IB-IIIA NSCLC patients, EGFR-TKIs adjuvant therapy could not only significantly improve DFS (HR 0.43, 95% CI 0.30–0.63, P < 0.001) and 2- and 3-year DFS rates, but also improve OS (HR 0.72, 95% CI, 0.54–0.96, P = 0.024), compared with chemotherapy or the placebo. Further subgroup analyses indicated prolonged OS from first-generation EGFR-TKI adjuvant therapy in stage III patients, compared with chemotherapy or the placebo (HR for OS, 0.34; 95% CI, 0.18–0.63; P = 0.001). Of note, osimertinib adjuvant therapy led to the OS benefit expanding from stage III to stage II-III patients, with significantly improved DFS and a lower risk of brain recurrence, compared with the placebo. A 2-year treatment duration with EGFR-TKI adjuvant therapy showed a significantly lower recurrence risk than a ≤ 1-year duration.

**Conclusion:**

The DFS advantage from first-generation EGFR-TKI adjuvant therapy can translate into an OS benefit in stage III NSCLC patients. Osimertinib might be more suitable for adjuvant therapy than first-generation EGFR-TKIs, because of the lower recurrence rate and the potential OS benefit even in early-stage patients. The optimal treatment duration for EGFR-TKIs at different stages of disease needs to be validated.

**Supplementary Information:**

The online version contains supplementary material available at 10.1186/s12885-023-11194-6.

## Background

Surgery is the most effective treatment for patients with resectable non-small-cell lung cancer (NSCLC), including those with a positive oncogenic driver such as epidermal growth factor receptor (EGFR) mutations. Compared with surgery alone, adjuvant platinum-based chemotherapy can further reduce the risk of disease recurrence and prolong survival in patients with stage II-IIIA or partial high recurrence risk stage IB NSCLC after complete resection [1]. However, adjuvant therapy is only able to decrease the risk of recurrence or death by 16%, yielding a 5.4% increase in 5-year overall survival (OS) [2], while it induces substantial treatment-related toxicity [3]. Therefore, it is necessary to seek alternative treatments with improved survival outcomes and tolerability, especially for stage III NSCLC patients with poor prognoses.

The success achieved with EGFR tyrosine kinase inhibitors (EGFR-TKIs) in advanced NSCLC patients has promoted the investigation of EGFR-TKIs as adjuvant treatment for resected EGFR-mutant NSCLC patients [4, 5]. To date, several clinical trials have investigated the feasibility of EGFR-TKIs when applied in an adjuvant setting. Although the results from the phase III BR19 trial demonstrated no superiority of gefitinib adjuvant treatment over the placebo in EGFR mutation-unselected patients in terms of either disease-free survival (DFS) or OS [6], several subsequent randomized controlled trials (RCTs) have shown that 2-year adjuvant therapy with first-generation EGFR-TKIs (such as gefitinib, erlotinib, or icotinib) can significantly improve DFS compared with chemotherapy or a placebo in patients with EGFR-mutant stage IB-IIIA NSCLC [7–10]. In addition, the phase 3 RCT ADAURA [11, 12] assessed the efficacy and safety of 3-year osimertinib treatment versus a placebo in patients with completely resected stage IB-IIIA EGFR-mutant NSCLC and showed the dramatic benefit in this setting with a 4-year DFS rate of 73% and a 5-year OS rate of 88%.

However, the long-term survival results of these trials are not consistent. Two phase III RCTs (ADJUVANT and IMPACT) that reported their final OS analysis at the American Society of Clinical Oncology (ASCO) Annual Meeting 2021 showed that adjuvant gefitinib appeared to prevent early relapse in patients with completely resected stage II-III EGFR-mutant NSCLC but did not significantly prolong OS in these patients [13, 14]. The phase II RCT EVAN showed that adjuvant erlotinib improved both DFS and OS in patients with EGFR-mutant stage IIIA NSCLC compared with chemotherapy [15]. Thus, whether adjuvant therapy with EGFR-TKIs can improve OS compared with chemotherapy and which stage of NSCLC can benefit from adjuvant EGFR-TKIs remains controversial. In addition, the types of adjuvant EGFR-TKI and the optimal treatment duration and regimen (EGFR-TKI alone or in combination with chemotherapy) are still unclear. The preliminary reports from the phase III RCT ADAURA showed that 3-year osimertinib treatment had superior DFS to the placebo regardless of stage, mutation type, or chemotherapy. Therefore, we conducted a meta-analysis of patients with resected EGFR-mutant NSCLC treated with adjuvant EGFR-TKIs versus chemotherapy or a placebo to address the questions relating to the application of adjuvant EGFR-TKIs in NSCLC patients.

## Methods

The present study was conducted according to the 2020 Preferred Reporting Items for Systematic Review and Meta-Analysis (PRISMA) guidelines [16]. The checklist of PRISMA 2020 for the meta-analysis is shown in Supplementary Table 1 (Table S1).

### Search strategy

The PubMed, Embase, Cochrane Library, and Chinese National Knowledge Infrastructure databases were searched systematically for relevant publications. The detailed search strategy of each database was shown in Supplementary Table 2 (Table S2). The following keywords were included: “lung cancer OR lung neoplasms OR lung carcinoma OR NSCLC OR non-small cell lung cancer” AND “adjuvant therapy” AND “epidermal growth factor receptor tyrosine kinase inhibitor OR EGFR-TKI OR erlotinib OR gefitinib OR icotinib OR afatinib OR dacomitinib OR almonertinib OR osimertinib” AND “randomized OR randomly”. The computer search was supplemented with manual searches of the references listed in all retrieved review articles, primary studies, and abstracts from meetings such as the ASCO, European Society for Medical Oncology (ESMO), and World Conference of Lung Cancer. The final literature search was performed on June 21, 2023.

### Study selection

Studies that met the following inclusion criteria were included in the meta-analysis: (1) phase II or III RCTs that compared the survival outcomes (DFS and/or OS) in stage I-IIIA NSCLC patients treated with adjuvant EGFR-TKIs versus adjuvant chemotherapy or a placebo or compared the efficacy for different durations of adjuvant EGFR-TKI therapy; (2) included patients who underwent primary radical resection harboring EGFR mutations (exon 19 deletion or exon 21 Leu858Arg); (3) hazard ratios (HRs) for survival analysis (DFS or OS) or the number of events for relevant clinical endpoint events from the overall patient population or subgroup analysis were available; and (4) contained original data sufficient to calculate HRs or P values. All of the included studies were published in English regardless of the publication status (published, conference proceedings, or unpublished).

The exclusion criteria were as follows: (1) letters, case reports, reviews, editorials, commentaries; duplicates, single-arm trials, observational studies, or nonclinical studies; (2) studies with irretrievable or insufficient data for statistical analysis; (3) studies written in languages other than English; and (4) low sample size (n < 10).

### Data extraction and quality assessment

For each included trial, we extracted the corresponding information, including the trial name, year of publication or conference presentation, first author, trial phase, number of participants, pathological stage, treatment regimen, generation of adjuvant EGFR-TKIs, and outcomes (such as survival and toxicity). Two of our authors (SLZ and LTH) extracted data and assessed the quality of the included studies using the Cochrane Risk of Bias (RoB) 2 tool [17] independently, and discrepancies were resolved by consensus.

### Statistical analysis

Data were analyzed using STATA 14.0 (STATA Corp, College Station, TX, USA) statistical software. For each eligible study, if the associated information was present merely in figures, two reviewers (SLZ and LTH) used Engauge Digitizer 10.8 software (produced by Mark Mitchell 2014; https://github.com/markummitchell/engauge-digitizer) to extract data from the statistical graphs independently. Data relating to DFS and OS were pooled and analyzed by HRs and 95% confidence intervals (CIs) (HR for disease progression or death in DFS, and HR for death in OS); data relating to survival rates and adverse events (AEs) were pooled and analyzed by relative risk (RR) and 95% CIs; and P values of less than 5% (P < 0.05) were considered statistically significant. I^2^ tests were used to test for heterogeneity, and no heterogeneity was assumed if P > 0.1 or I^2^ < 50% using the fixed-effects model (Mantel–Haenszel method). Otherwise, a random-effect model (DerSimonian–Laird method) was applied. In addition, sensitivity analysis for the primary outcomes was conducted based on the leave-one-out approach, and publication bias was evaluated by a funnel plot and Begg’s and Egger’s tests.

## Results

### Literature review and characteristics of the included studies

**Fig. 1:**
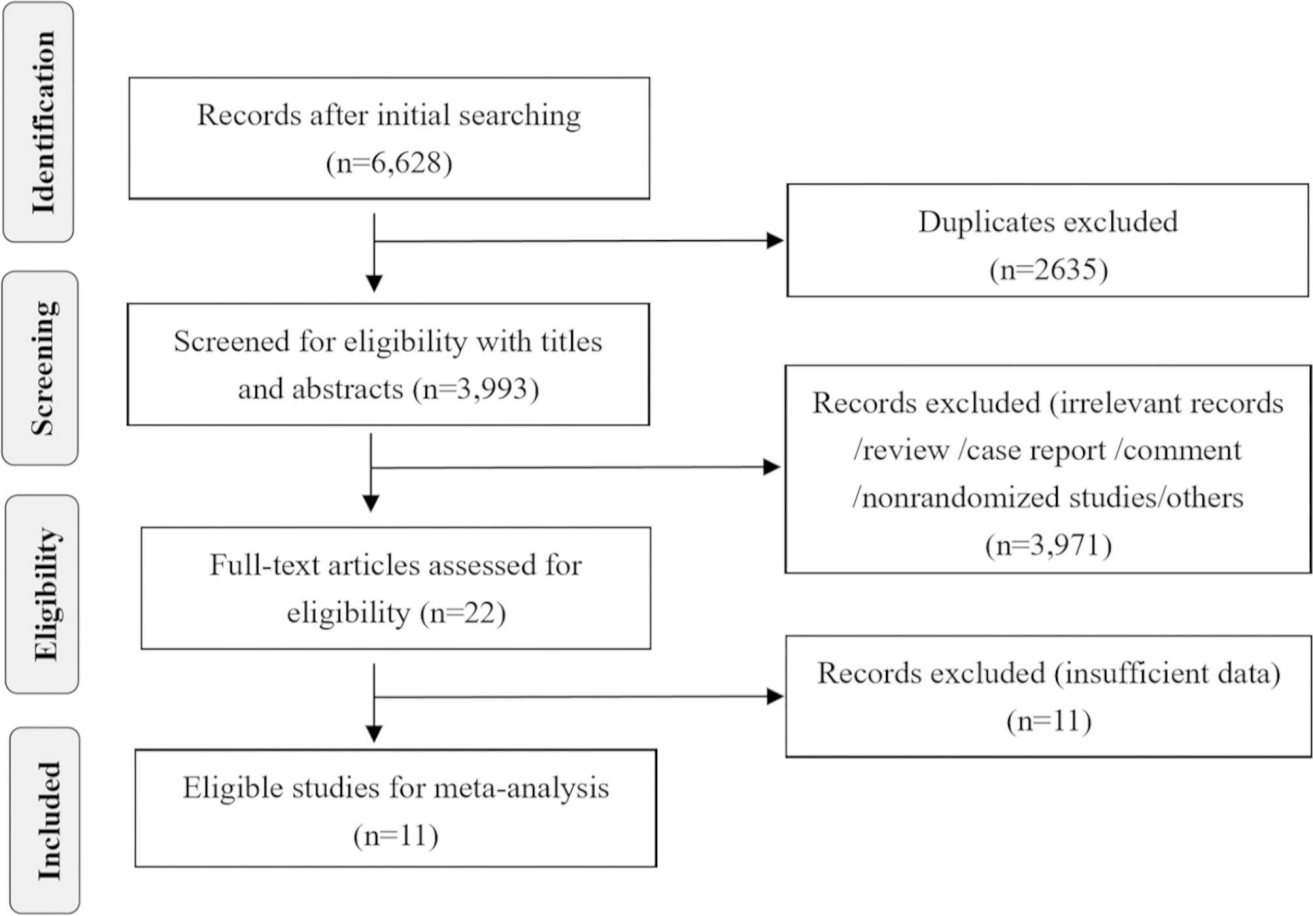
Overview of the study search and selection

A PRISMA flow diagram of the literature search process is shown in Fig. 1. Overall, 6628 records were identified according to the search strategy, and 6606 records were excluded after screening the titles and abstracts. Of the remaining 22 potentially relevant studies, 11 were excluded because of a lack of survival data. Finally, 11 RCTs involving 2102 patients harboring EGFR mutations were included in the present meta-analysis according to the search strategy. In detail, the literature search yielded 11 eligible RCTs, including five RCTs that compared adjuvant first- or third-generation EGFR-TKIs with a placebo, four RCTs that compared adjuvant first-generation EGFR-TKIs with chemotherapy, and two RCTs that compared the duration of 2-year adjuvant first- or second-generation EGFR-TKI treatment with ≤ 1-year treatment. Table 1 shows a summary of the patient, tumor, and treatment characteristics for each trial [7–11, 13–15, 18–20].

**Table 1:** Characteristics of the trials included in the meta-analysis

Trials (year)	Phase	Intervention	TKI treatment duration (month)	Stage	No. of patients	Adjuvant chemotherapy Yes/No (No.)	Outcomes
Li et al. (2014)	II	PC-gefitinib	6	IIIA (N2)	30	30/0	DFS, OS
		PC		IIIA (N2)	30	30/0	
RADIANT (2015)	III	Erlotinib	24	IB-IIIA	102	46/56	DFS, OS, safety
		Placebo		IB-IIIA	59	23/26	
Feng et al. (2015)	II	Chemotherapy + Icotinib	4–8	IB	9	9/0	DFS
				II	5	5/0	
				IIIA	7	7/0	
		Chemotherapy		IB	8	8/0	
				II	5	5/0	
				IIIA	5	5/0	
ADAURA (2023)	III	Osimertinib	36	IB	107	27/80	DFS, OS, safety
				II	115	80/35	
				IIIA	117	95/22	
		Placebo		IB	109	30/79	
				II	116	85/31	
				IIIA	118	92/26	
EVIDENCE (2021)	III	Icotinib	24	II–IIIA	161	0/161	DFS, OS, safety
		Chemotherapy		II–IIIA	161	161/0	
ADJUVANT (2021)	III	Gefitinib	24	II-IIIA	111	0/111	DFS, OS, safety
		VP		II-IIIA	111	111/0	
IMPACT (2022)	III	Gefitinib	24	II	42	0/42	DFS, 5-year OS rate
				III	74	0/74	
		VP		II	44	44/0	
				III	72	72/0	
EVAN (2022)	II	Erlotinib	24	IIIA	51	0/51	DFS, OS, safety
		VP		IIIA	51	51/0	
CORIN (2023)	II	Icotinib	12	IB	63	0/63	DFS, safety
		Placebo		IB	65	0/65	
Neal et al. (2021)	II	Afatinib	3	I-III	23	12/11	DFS, 2-year recurrence rate, safety
		Afatinib	24	I-III	22	10/12	
ICOMPARE (2023)	II	Icotinib	12	II-IIIA	55	0/55	DFS, safety
		Icotinib	24	II-IIIA	54	0/54	

Note: TKI, tyrosine kinase inhibitor; DFS, disease-free survival; OS, overall survival; PC, pemetrexed plus carboplatin; VP, vinorelbine plus cisplatin

### DFS benefit with adjuvant EGFR-TKIs

#### All patients

**Fig. 2:**
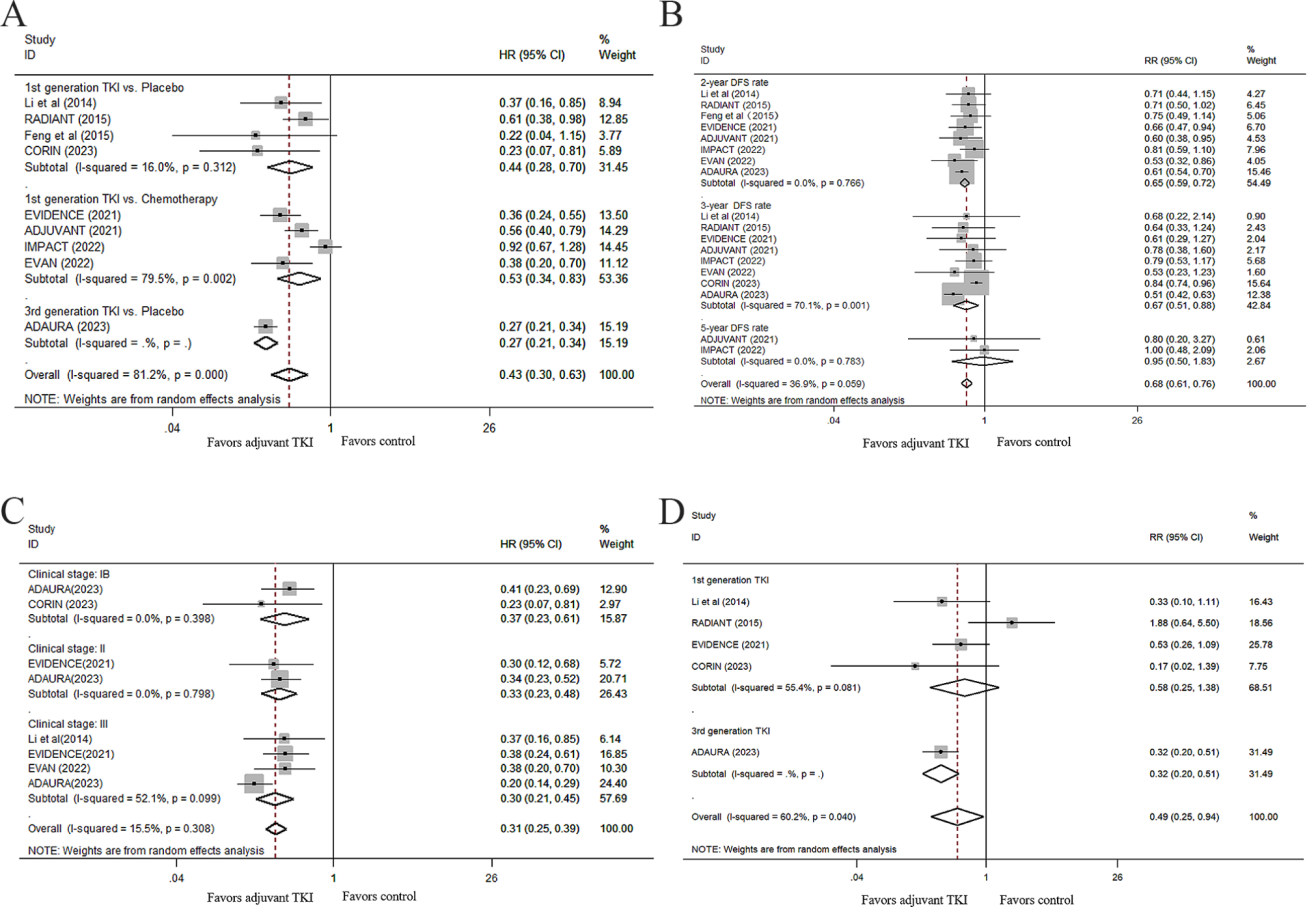
Effects of adjuvant EGFR-TKI on DFS. Forest plot of the HRs of DFS for adjuvant TKI vs. non-TKI therapy for resected patients with IB-III stage EGFR-mutant NSCLC (**A**), and for patients with different stages of disease (**C**); Forest plot of the RRs of the 2, 3, and 5-year DFS rates (**B**) and brain relapse rates (**D**) for adjuvant TKI vs. non-TKI therapy in these patients. EGFR-TKI, epidermal growth factor receptor tyrosine kinase inhibitor; DFS, disease-free survival; HR, hazard ratio; RR, relative risk; NSCLC, non-small-cell lung cancer

Overall, nine studies (1948 patients) were included in the analysis of DFS for the comparison between the EGFR-TKI group (TKI only/TKI plus chemotherapy) and the non-EGFR-TKI (control) group (placebo/chemotherapy) in an adjuvant setting for patients with resected EGFR-mutant NSCLC. As shown in Fig. 2A, heterogeneity was detected after applying the heterogeneity test (I^2^ = 81.2%); thus, a random-effects model was applied. Our results showed that adjuvant therapy with EGFR-TKIs compared with non-EGFR-TKIs was significantly associated with a 57% reduction in the risk of disease progression or death (HR, 0.43; 95% CI, 0.30–0.63; P < 0.001). Also, a significant reduction in the risk of disease progression or death (HR, 0.49; 95% CI, 0.35–0.68; P < 0.001; Supplementary Fig. 1 (Fig. S1)) was found in patients treated with first-generation EGFR-TKIs when the ADAURA trial was excluded. We further investigated the effect of EGFR-TKIs on 2-, 3-, and 5-year DFS rates. The pooled results showed that EGFR-TKI adjuvant therapy improved the 2- and 3-year DFS rates significantly compared with those without TKI treatment (RR, 0.65 and 0.67; P < 0.001 and P = 0.004, respectively; Fig. 2B). When we excluded the ADAURA trial, although the superiority of first-generation EGFR-TKIs on 2- and 3-year DFS rates remained (RR, 0.69 and 0.81; P < 0.001 and P = 0.001, respectively; Fig. S1), no significant advantage was observed in the 5-year DFS rate for the first-generation EGFR-TKIs in EGFR-mutant NSCLC patients (RR, 0.95; P = 0.886; Fig. 2B).

### Subgroup analysis

A series of subgroup analyses were performed to explore the effects of variables on the efficacy of EGFR inhibitors in resected EGFR-mutant NSCLC patients. The subgroup included interventions (adjuvant EGFR-TKI versus placebo or chemotherapy), different generations of EGFR-TKIs, clinical stage, brain recurrence and EGFR mutation status (exon 19 deletion and L858R mutation).

To further explore the effects of different interventions on DFS, subgroup analyses of DFS in patients treated with adjuvant EGFR-TKIs vs. a placebo and in those treated with adjuvant EGFR-TKIs vs. chemotherapy were performed. Five RCTs compared adjuvant first-generation EGFR-TKIs with a placebo. The ADAURA study compared the adjuvant third-generation EGFR-TKI osimertinib with a placebo. Four other studies compared adjuvant chemotherapy followed by first-generation EGFR-TKIs (gefitinib/erlotinib/icotinib) with adjuvant chemotherapy alone or a placebo [7–9, 18]. The DFS of patients treated with EGFR-TKIs was superior to that of patients treated with a placebo (P = 0.001), especially for the third-generation EGFR-TKI osimertinib, which corresponded to a 73% reduction in the risk of disease progression or death (Fig. 2A). The other four RCTs compared adjuvant EGFR-TKI therapy with adjuvant chemotherapy. The results demonstrated that adjuvant EGFR-TKI therapy also resulted in better DFS for resected EGFR-mutant NSCLC patients than adjuvant chemotherapy, with a HR of 0.53 (95% CI, 0.34–0.83; P = 0.006; random effect, I^2^ = 79.5%; Fig. 2A).

Data on DFS based on stage IB, II, or III disease were available in five RCTs (Fig. 2C) and the pooled results suggested a significant improvement in DFS with EGFR-TKIs in stage IB, II and III NSCLC patients, respectively. In addition, we analyzed the effect of EGFR-TKI adjuvant treatment on the DFS of patients with stage II-III NSCLC. Our results demonstrated that EGFR-TKIs showed a significant beneficial effect on DFS in these patients (HR, 0.43; 95% CI, 0.26–0.71; P = 0.001; Supplementary Fig. 2 (Fig. S2)).

Based on the available data on the brain recurrence rate reported from five trials (ADAURA, Li’s study, RADIANT, EVIDENCE and CORIN), an analysis of the effects of EGFR-TKIs on brain recurrence was conducted. EGFR-TKI therapy reduced the risk of brain recurrence (RR, 0.49; 95% CI, 0.25–0.94; P = 0.031; random effect, I^2^ = 60.2%; Fig. 2D). Of note, when we excluded the ADAURA trial, the superiority of EGFR-TKIs disappeared. Thus, adjuvant first-generation EGFR-TKIs were not superior to the placebo in reducing brain recurrence (RR, 0.58; 95% CI, 0.25–1.38; P = 0.217; random effect, I^2^ = 55.4%; Fig. 2D).

The results from subgroup analyses of DFS in patients with exon 19 deletion versus L858R mutation indicated that EGFR-TKI treatment had a favorable effect on DFS in patients harboring either exon 19 deletion (HR, 0.41; 95% CI, 0.26–0.65; P < 0.001; random effect, I^2^ = 74.1%; Supplementary Fig. 2 (Fig. S2)) or L858R mutation (HR, 0.52; 95% CI, 0.36–0.77; P = 0.001; random effect, I^2^ = 62.6%; Fig. S2). No significant difference in DFS was observed between the EGFR exon 19 deletion and L858R mutation subgroups (P for heterogeneity = 0.051).

### OS benefit with adjuvant EGFR-TKIs

**Fig. 3:**
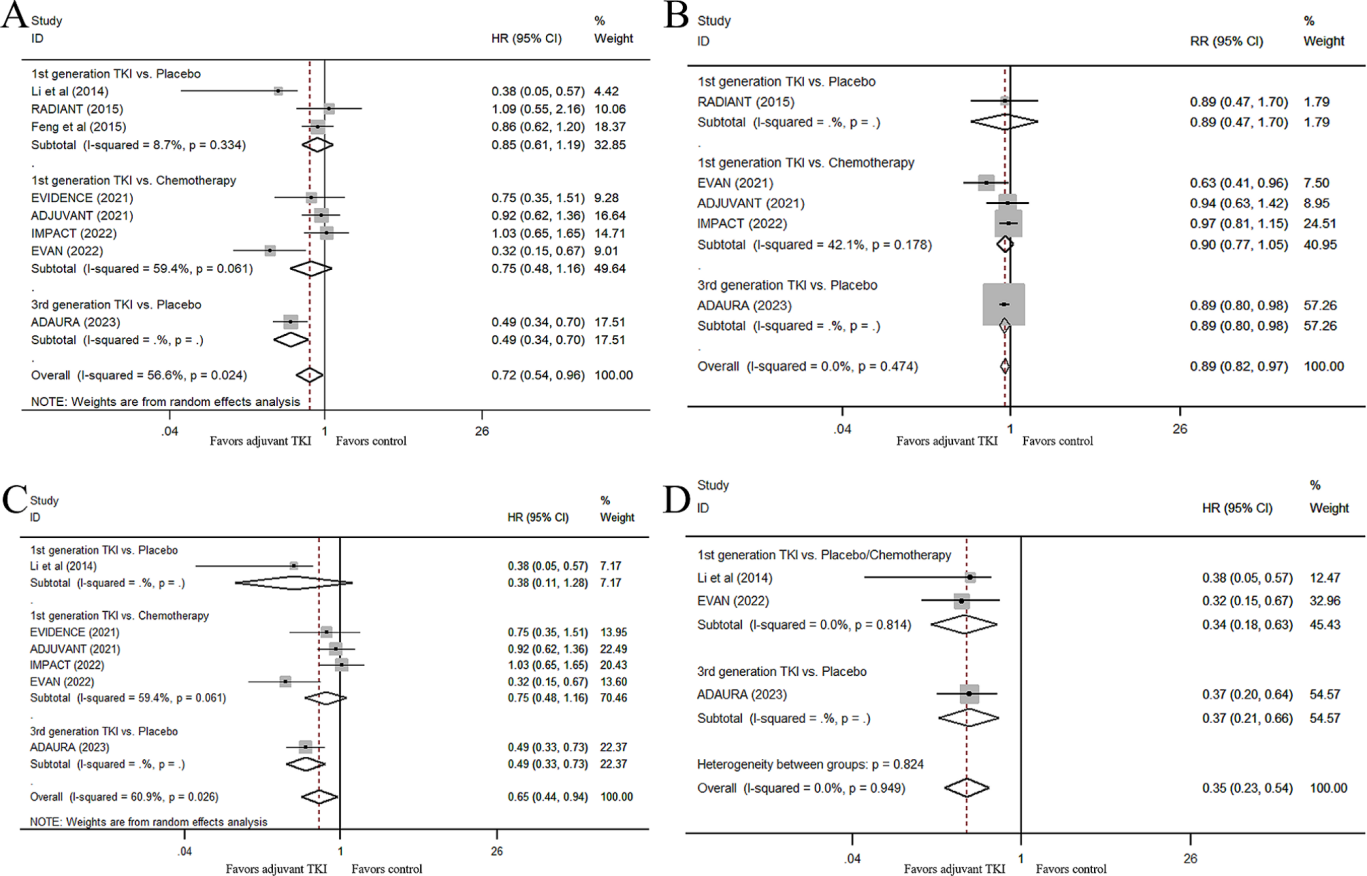
Effects of adjuvant EGFR-TKI on OS. Forest plot of the HRs of OS for adjuvant TKI vs. non-TKI therapy for resected patients with IB-III stage EGFR-mutant NSCLC (**A**), for all stage II-III patients (**C**) and for stage III patients (**D**); Forest plot of the RRs for the 5-year OS rates (**B**) between different groups. EGFR-TKI, epidermal growth factor receptor tyrosine kinase inhibitor; OS, overall survival; HR, hazard ratio; RR, relative risk; NSCLC, non-small-cell lung cancer

For all patients with stage IB-III NSCLC, the pooled OS analysis from eight RCTs with available or updated data showed a significant improvement in long-term survival in the adjuvant EGFR-TKI group compared with the control group in resected EGFR-mutant NSCLC (HR, 0.72; 95% CI, 0.54–0.96, P = 0.024, Fig. 3A). However, the OS improvement from EGFR-TKIs was not observed when we excluded the ADAURA trial, indicating that adjuvant first-generation EGFR-TKIs were not superior to placebo/chemotherapy in prolonging OS in these patients (HR, 0.80; 95% CI, 0.61–1.05, P = 0.103, Supplementary Fig. 3 (Fig. S3)). No improvement could be found in the subgroup analysis involving the comparison of the adjuvant first-generation EGFR-TKI group with the chemotherapy group (HR, 0.75; 95% CI, 0.48–1.16, P = 0.189, Fig. 3A), or the placebo group (HR, 0.85; 95% CI, 0.61–1.19; P = 0.345; Fig. 3A) in resected EGFR-mutant NSCLC. Additionally, we also pooled the available data of 5-year OS rates between EGFR-TKI group and non-EGFR-TKI group for patients with stage IB-III NSCLC, an improvement of 5-year OS rate could be seen in the EGFR-TKI group (RR, 0.89, 95% CI, 0.82–97, P = 0.008, Fig. 3B), while the difference in the 5-year OS rate between the two groups was not significant when we included the ADAURA trial (RR, 0.90, 95% CI, 0.77–1.04, P = 0.162, Fig. S3).

We further investigated the effect of EGFR-TKI adjuvant treatment on the OS of patients with stage II-III NSCLC based on the extraction data of the included studies. The results showed that EGFR-TKI adjuvant treatment could significantly reduce the risk of death in patients with stage II-III NSCLC (HR, 0.65; 95% CI, 0.44–0.94; P = 0.022; Fig. 3C), when the ADAURA study was included. Although there was a 30% reduction in the risk of death in patients treated with first-generation EGFR-TKI adjuvant treatment compared with patients treated with adjuvant chemotherapy or the placebo (HR, 0.70; 95% CI, 0.46–1.07; P = 0.097; random effect, I^2^ = 54.7%; Fig. S3), the difference was not significant after exclusion of the ADAURA study. Next, we compared EGFR-TKI adjuvant therapy versus non-EGFR-TKI treatment according to clinical stage. Because only limited data could be extracted from the included study, the OS HR values in patients with stage II NSCLC were not available. Thus, we only used the pooled HRs of OS in patients with stage III NSCLC. As shown in Fig. 3D, compared with the non-EGFR-TKI treatment group, adjuvant EGFR-TKI treatment could significantly prolong the OS in EGFR-mutant patients with stage III NSCLC, with a HR of 0.35 (95% CI, 0.23–0.54, P < 0.001). Also, first-generation EGFR-TKI treatment significantly reduced the risk of death in these patients with stage III NSCLC (HR, 0.34; 95% CI, 0.18–0.63, P = 0.001, Fig. 3D), when we excluded the ADAURA trial.

### Subsequent EGFR-TKI treatment

**Fig. 4:**
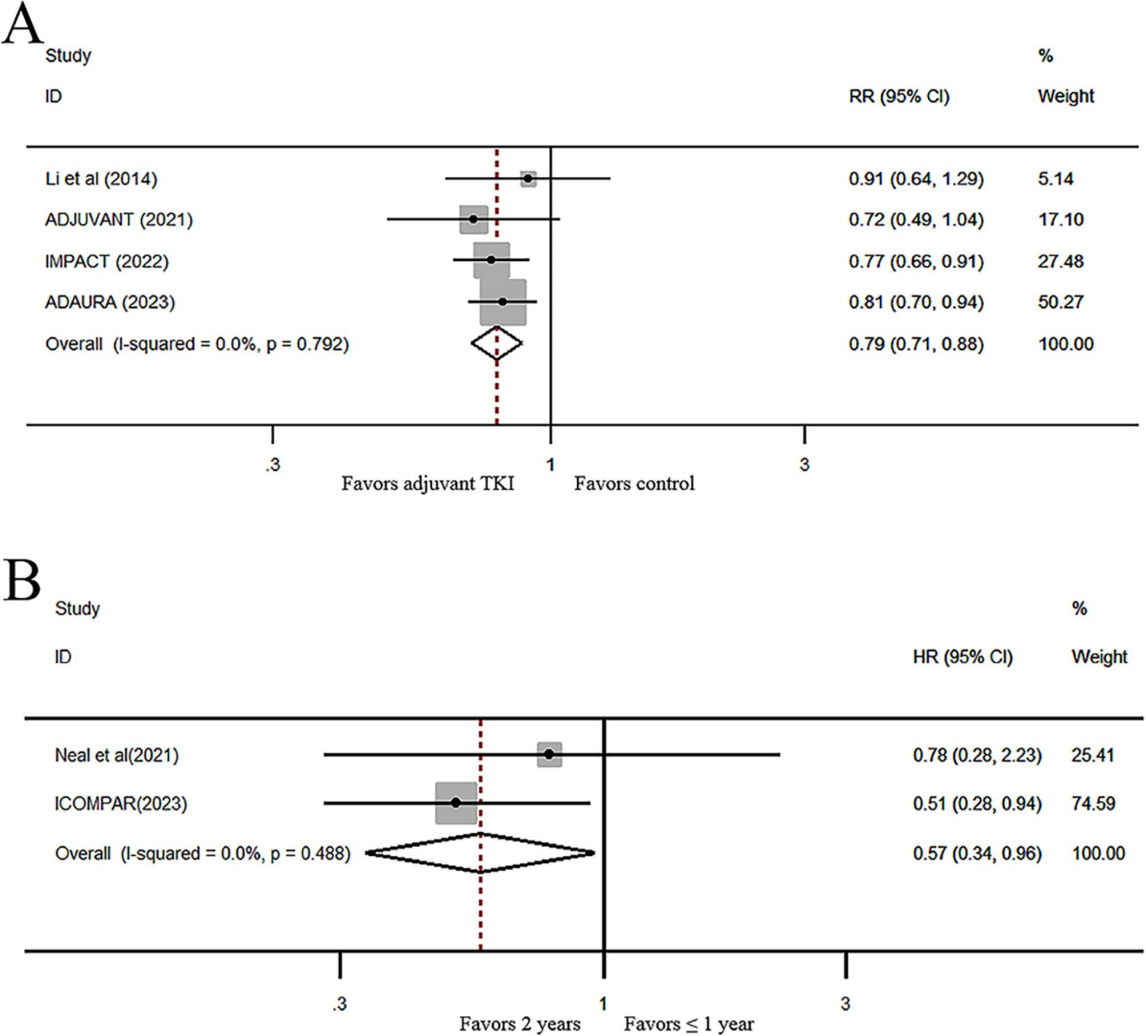
Forest plot of RRs for the rates of subsequent EGFR-TKI treatment (**A**) and HRs of DFS for different durations of adjuvant therapy (**B**) in resected patients with EGFR-mutant NSCLC. RR, relative risk; EGFR-TKI, epidermal growth factor receptor tyrosine kinase inhibitor; DFS, disease-free survival; HR, hazard ratio; EGFR, epidermal growth factor receptor

Three RCTs reported subsequent EGFR-TKI treatment, including different generation TKIs. There were more patients in the control group (80.0%; 95% CI, 65.5–94.4%) who received subsequent EGFR-TKI therapy than in the EGFR-TKI adjuvant treatment group (63.4%; 95% CI, 44.6–82.2%), with an RR value of 0.79 (95% CI, 0.71–0.88; P < 0.001; Fig. 4A).

### The impact of different durations of EGFR-TKI adjuvant therapy on survival outcomes

Only two RCTs compared the DFS of patients who received adjuvant first- or second-generation EGFR-TKI therapy for ≤ 1 year (1 year in one study, 3 months in another study) with those who received first- or second-generation EGFR-TKI therapy for 2 years [19, 20]. Although the OS data were not yet complete, the pooled result suggested a significantly lower risk of recurrence after 2 years of adjuvant treatment with first- or second-generation EGFR-TKIs than after ≤ 1 year of adjuvant treatment in patients with stage IB-IIIA NSCLC-positive EGFR mutations (HR, 0.57; 95% CI, 0.34–0.96; P = 0.034; Fig. 4B).

### Grade ≥ 3 treatment-related AEs

**Fig. 5:**
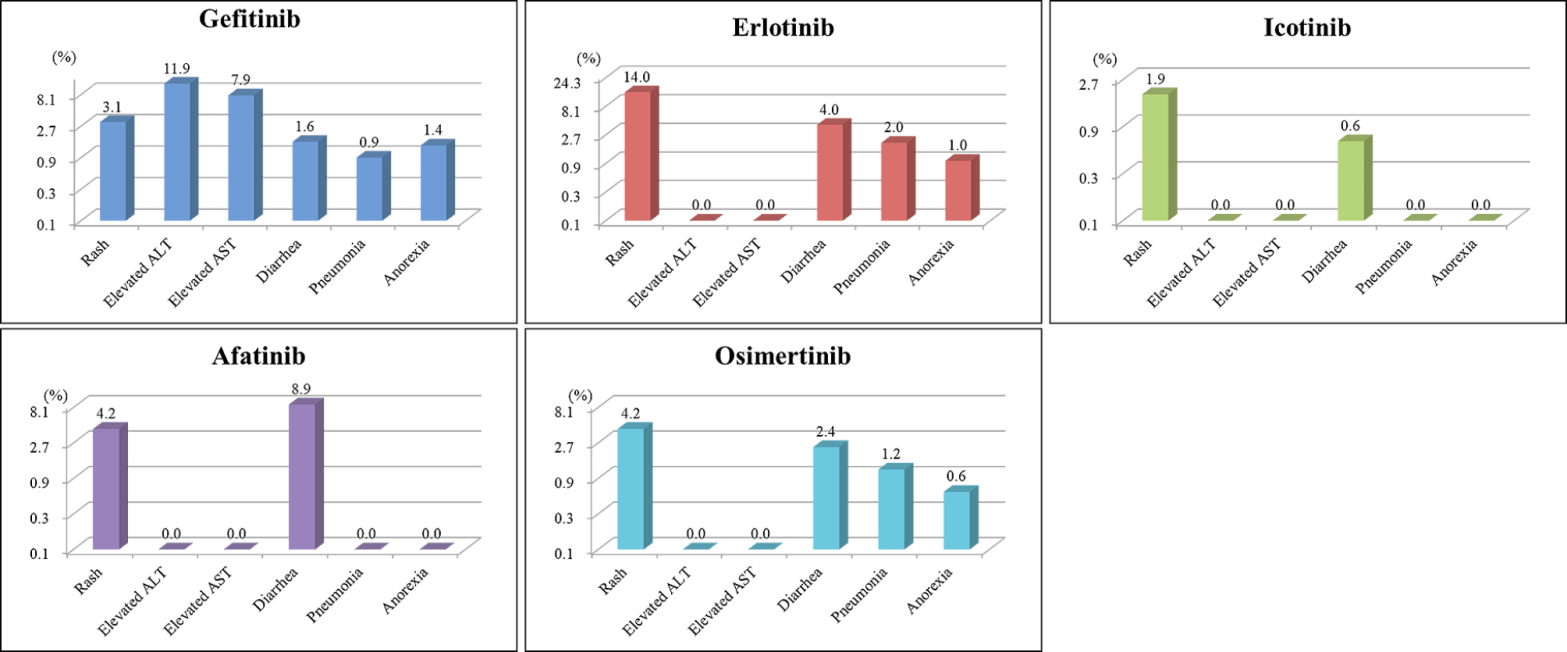
Grade ≥ 3 AEs caused by different EGFR-TKI treatments

The most common severe AEs (grade ≥ 3) documented in 10 of the 11 selected studies, including 1064 EGFR-mutated patients treated with EGFR-TKIs, were elevated alanine transaminase (11.9%, 95% CI, 0.0–32.3%), elevated aspartate transaminase (7.9%, 95% CI, 0.0–20.6%), rash (4.3%; 95% CI, 1.6–6.9%), stomatitis (1.8%, 95% CI, 0.4–3.2%), diarrhea (1.7%; 95% CI, 0.8–2.7%), pneumonia (1.2%, 95% CI, 0.2–2.1%), anorexia (0.7%, 95% CI, 0.0–1.4%), dry skin (0.4%, 95% CI, 0.0–0.9%), and asthenia (0.4%, 95% CI, 0.0–0.9%). The pooled data of EGFR-TKI-related grade ≥ 3 AEs are shown in Supplementary Table 3 (Table S3). In addition, we also analyzed the incidence rates of related grade ≥ 3 AEs, subgrouped as above, by different agents. As shown in Fig. 5, grade ≥ 3 elevated alanine transaminase (11.9%)/elevated aspartate transaminase (7.9%), rash (14%), and diarrhea (8.9%) commonly occurred in gefitinib-, erlotinib-, and afatinib-treated patients, respectively.

### Sensitivity analysis

**Fig. 6:**
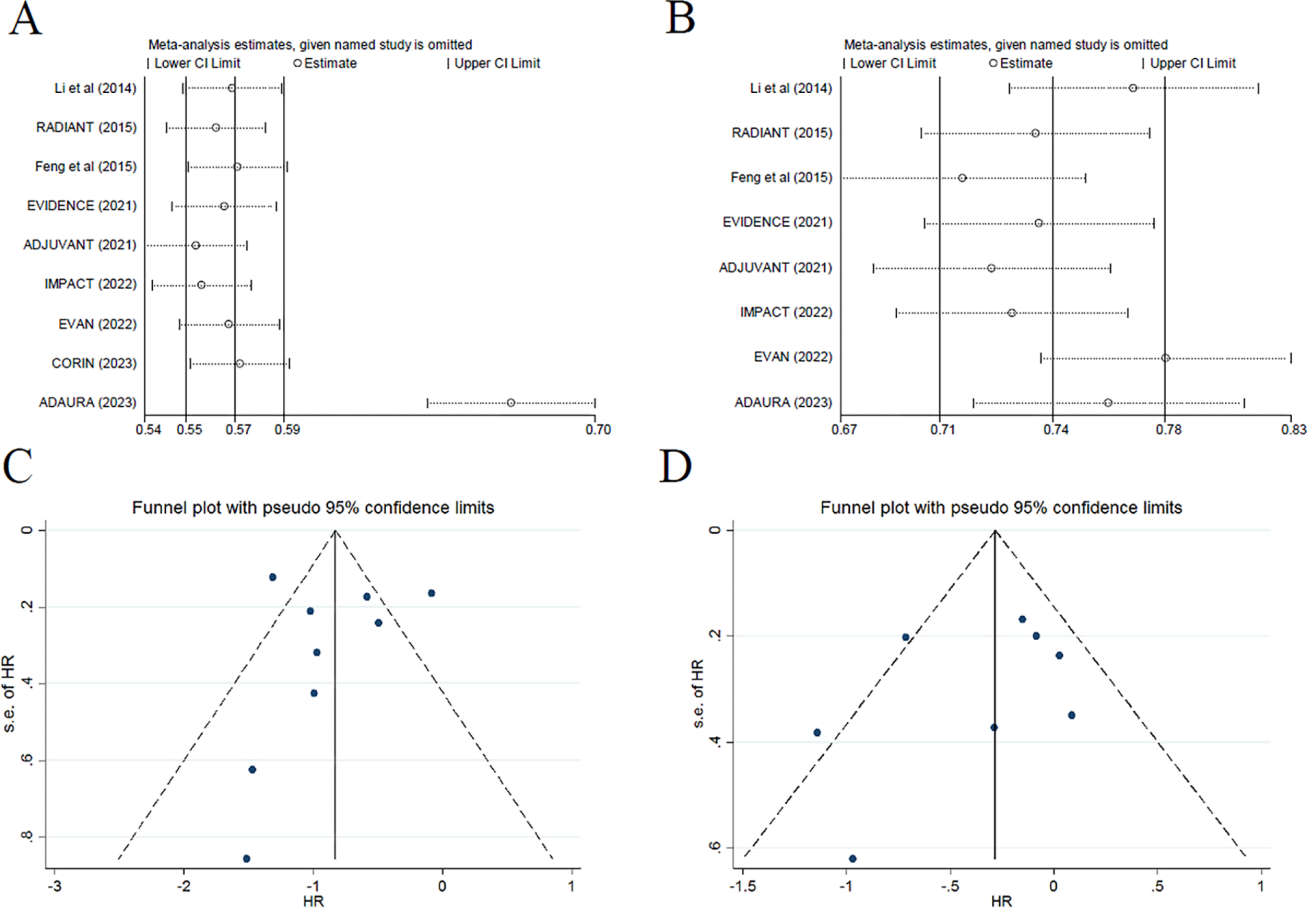
Sensitivity analysis for DFS (**A**) and OS (**B**) and publication bias assessment for DFS (**C**) and OS (**D**). DFS, disease-free survival; OS, overall survival

Sensitivity analysis was used to assess the impact of each individual study on the pooled HRs of the median DFS and OS by sequentially removing each eligible study. For DFS analysis, the HR value in each trial was similar to the pooled HR (0.57, 95% CI, 0.55–0.59), except for the ADAURA study, which resulted in a higher HR value when this study was omitted (0.67, 95% CI, 0.64–0.70; Fig. 6A). For OS analysis, the HR values were slightly higher than the pooled HR (0.74, 95% CI, 0.70–0.78) when the Li’s (HR, 0.77; 95% CI, 0.73–0.81), EVEN (HR, 0.78; 95% CI, 0.74–0.82) and ADAURA (HR, 0.76; 95% CI, 0.72–0.81) studies were excluded separately (Fig. 6B).

### Study quality and publication bias

As summarized in Supplementary Fig. 4 (Fig. S4), all trials were assessed as moderate-to-high quality. Minimal or no publication bias for DFS and OS was detected via funnel plots (Fig. 6C, D). Moreover, additional tests failed to find any publication bias for the outcome DFS (Egger’s test P = 0.466; Begg’s test P = 0.807) or OS (Egger’s test P = 0.536; Begg’s test P = 0.068).

## Discussion

Eleven RCTs evaluated the value of postoperative adjuvant EGFR-TKIs. Our meta-analysis showed that EGFR-TKI adjuvant therapy significantly improved DFS compared with adjuvant therapy without EGFR-TKIs, regardless of stage, generation of EGFR-TKI, or mutation type, along with an OS improvement despite heterogeneity in the presence of subgroup outcomes. Subgroup analysis showed that compared with non-EGFR-TKI treatment, OS improvement of first-generation EGFR-TKI adjuvant treatment was observed only in stage III NSCLC patients, but not in all stage IB-III patients. Additionally, a 2-year adjuvant treatment duration with first- or second-generation EGFR-TKIs can further reduce the recurrence risk compared with ≤ 1-year adjuvant treatment. The results on adjuvant therapy for third-generation EGFR-TKIs are limited to osimertinib, but the present analyses showed a significant prolongation of DFS as well as a substantial reduction in the risk of brain metastases, which ultimately translated into a statistically significant OS benefit.

Recently, some meta-analyses [21–24] have also suggested that EGFR-mutant NSCLC patients benefit from EGFR-TKI adjuvant therapy after radical resection. However, those meta-analyses only included RCTs or retrospective studies up to 2020, and the survival results including OS and DFS in the ADJUVANT, EVIDENCE, IMPACT, EVAN, CORIN, ADAURA trials reported in 2021 to 2023 were not included in those meta-analyses. Our meta-analysis included four large RCTs (EVIDENCE, ADJUVANT, ICOMPARE, and Neal’s study), two previous studies with updated survival data in 2022 (EVAN and IMPACT trials), CORIN trial with updated survival data in 2023, and ADAURA trial with updated DFS and OS data in 2023. These high-quality studies further strengthened the evidence for the efficacy of EGFR-TKI adjuvant therapy, and also complemented the results lacking in the above meta-analysis. Two recently published meta-analysis [25, 26] (neither of which included updated data of CORIN, ICOMPARE, Neal’s study, EVAN and ADAURA) did not define whether adjuvant therapy with EGFR-TKIs could prolong OS in these patients. In addition to the lack of the latest updated data on DFS and OS, some important outcomes that could further demonstrate the superiority of EGFR-TKI adjuvant therapy, such as DFS/OS rates at different time points, OS improvement at different stages, and the possible impact of subsequent treatment on OS, were not evaluated in these meta-analysis. In our present analysis, we draw the conclusion that EGFR-TKI adjuvant therapy can improve OS through including the new trials and updated OS data, thus providing a clear answer to the question of whether adjuvant therapy with EGFR-TKIs can lead to improved OS, which was not clearly answered in previous studies, and further emphasizing the role of EGFR-TKI adjuvant therapy in resected EGFR-mutant NSCLC patients. In addition, our meta-analysis was the first to explore the optimal adjuvant treatment duration by including two RCTs, ICOMPARE and Neal’s study. This can be considered as add-on value compared to those previous reports.

First, we will focus on the benefits for DFS, as DFS was the primary endpoint of all of the included studies. Although the benefit of EGFR-TKI adjuvant therapy in terms of DFS has been defined, the optimal regimen and generation of EGFR-TKI for patients remained to be identified. Similar to previous studies [21–23], the present meta-analysis results demonstrated that EGFR-TKI treatment of EGFR-mutant NSCLC patients improved DFS compared with non-EGFR-TKI treatment in an adjuvant setting after radical resection. We further pooled the DFS data by subgrouping through different types of EGFR-TKIs as well as comparisons with different interventions. Of note, osimertinib treatment exhibited significantly longer DFS and a greater reduction in the brain recurrence risk than first-generation EGFR-TKIs (gefitinib/erlotinib/icotinib). To gain further insight into adjuvant chemotherapy use and its impact on efficacy outcomes in resected NSCLC, a recent study that analyzed data from the ADAURA trial indicated that the adjuvant osimertinib was an effective treatment for patients with stage IB-IIIA EGFR-mutant NSCLC after resection, with or without prior adjuvant chemotherapy [11, 27]. Although patients in the ADAURA trial were not randomized to compare adjuvant osimertinib versus adjuvant chemotherapy +/˗ osimertinib, the preliminary results did not demonstrate that adjuvant chemotherapy was harmful in the context of resected EGFR-mutant NSCLC. Whether adjuvant chemotherapy combined with osimertinib could prolong survival compared with adjuvant osimertinib alone requires further study. Additionally, we also investigated the DFS benefits in resected patients based on the type of EGFR mutation, and no significant difference was observed between the exon 19 deletion and the L858R mutation.

The present study also analyzed the effect of EGFR-TKI on the rate of DFS to better compare the long-term cure rate associated with adjuvant therapy. The findings showed that adjuvant therapy with first-generation EGFR-TKIs led to significantly improved 2- and 3-year DFS rates, but there was no significant improvement in the 5-year DFS rate, compared with non-EGFR-TKI treatment for patients with resected EGFR-mutant NSCLC. There are several possible reasons to explain the failure of first-generation EGFR-TKIs in long-term DFS improvement: (1) the use of first-generation EGFR-TKIs may induce some biological behavior changes in NSCLC patients, resulting in the induction of drug resistance mechanisms [28]; (2) the limited treatment duration of first-generation EGFR-TKIs may be another reason. To date, the optimal duration time of adjuvant EGFR-TKI treatment has not been clearly defined. Although our present study showed that 2-year adjuvant treatment with first- or second-generation EGFR-TKIs was superior to a duration of less than 1 year in terms of DFS benefits, only two RCTs [19, 20] have been published to date that involve a comparison of treatment duration between 2 years and ≤ 1 year, and data on treatment durations of more than 2 years are lacking. The median durations of first-generation EGFR-TKI treatment were 21.9, 11, and 6 months for the ADJUVANT [13], RADIANT [8], and Li trials [7], respectively. In the ADJUVANT trial, the Kaplan–Meier curves for DFS in the gefitinib group versus the chemotherapy group separated at 12 months and converged again at 36 months, suggesting an approximate 12-month therapeutic benefit for patients treated with gefitinib after TKI termination at 24 months. In the EVAN study, patients treated with erlotinib after a median EGFR-TKI treatment duration of 23.9 months maintained a benefit for at least 24 months after TKI termination at 24 months and achieved improved OS. Thus, a longer duration of TKI treatment might be the major reason for the low rate of recurrence and promising OS, although mature data with a duration of more than 2 years are still lacking.

It is worth noting whether the DFS advantage can be translated to a significant improvement in OS because this can determine the ultimate value of adjuvant therapy with EGFR-TKIs. The present meta-analysis suggested that first-generation EGFR-TKI adjuvant therapy can improve OS in patients with stage III EGFR-mutant NSCLC, but had no impact on OS for stage IB-II NSCLC patients. The lack of OS benefit may be due to several reasons.

First, the minor DFS gap between prior adjuvant EGFR-TKIs and chemotherapy/placebo may not correlate with a statistically significant OS difference. In theory, longer EGFR-TKI treatment or a more potent EGFR-TKI may increase this difference. As the results reported in ICOMPARE study [20] suggested, a longer duration of TKI treatment might contribute to a prolonged OS in stage II-III EGFR-mutant resected NSCLC patients. Although adjuvant EGFR-TKIs showed a significant DFS improvement in each of the included trials in the present study, the separated duration of the Kaplan-Meier curves for DFS after TKI termination in each treatment group varied among different trials. As mentioned above, an about 12-month and an at least 24-month therapeutic benefit was observed for patients in first-generation EGFR-TKI treatment group after TKI termination at 24 months in ADJUVANT trial [13] and EVAN trial [15], respectively. Compared with early-generation EGFR-TKIs, osimertinib may be more effective for adjuvant treatment, because it can permanently reduce disease recurrence and significantly improve OS. In ADAURA trial [12], the Kaplan–Meier curves for DFS in the osimertinib group versus the control group separated at 3 months and exhibited an absolute difference of 41% in 3-year DFS rate between the two groups. In addition, the two Kaplan–Meier curves for DFS in ADAURA trial have not converged again at 66 months, indicating an at least 30-month therapeutic benefit for patients treated with osimertinib after TKI termination at 36 months. A five-state semi-Markov model that predicted improved OS with osimertinib versus the placebo in an adjuvant setting, suggested that the adjuvant osimertinib may increase life expectancy significantly in patients with stage IB-IIIA EGFR-mutant NSCLC compared with the placebo, with a 17.4% absolute improvement in OS at 10 years [29]. In fact, the pooled OS data for stage IB-IIIA NSCLC patients in the present meta-analysis also indicated a strong OS benefit from osimertinib adjuvant treatment (Fig. 3A), thus strengthening the evidence for the efficacy of adjuvant osimertinib treatment in terms of OS in patients with EGFR-mutant NSCLC.

Second, different follow-up treatments in different groups may narrow the OS gap. Our pooled data from four RCTs [7, 11, 13, 14] regarding follow-up treatment showed that more patients in the non-TKI group received a variety of subsequent EGFR-TKI treatments than those in the EGFR-TKI group (RR, 0.79; P < 0.001). These unbalanced subsequent treatments might be one of the possible reasons to explain the similar OS in the two groups when subgrouped by comparison of the adjuvant EGFR-TKI group with the chemotherapy/placebo group. In the ADJUVANT trial [13], 36.8% (28/76) and 51.5% (35/68) of patients in the gefitinib arm and chemotherapy arm, respectively, received subsequent treatment, including targeted (any TKI) therapy alone or in combination with chemotherapy or local treatment. The median OS in patients with disease progression who received subsequent treatment in the gefitinib group and chemotherapy group was 57.4 and 51.9 months, respectively; the median OS in those who did not receive subsequent treatment was only 28.7 and 15.6 months in the two groups, respectively. This suggests that EGFR-mutant lung cancers retain sensitivity to EGFR-TKIs upon retreatment; subsequent targeted therapy after failure of adjuvant targeted therapy may provide longer OS and may be a better sequential treatment model for patients with resected EGFR mutation NSCLC. Furthermore, in the IMPACT trial, more patients in the chemotherapy group received subsequent targeted (any TKI) therapy than in the gefitinib group (93.5% vs. 72.3%). In the ADAURA trial [11], 22% patients in the osimertinib group and 54% patients in the placebo group had received subsequent anticancer treatments (the most commonly used treatment was EGFR-TKIs, most frequently osimertinib). Although this may have reduced the difference in OS seen between the osimertinib and placebo group, the ultimate OS benefit remained dominant. It suggested that subsequent targeted therapy in the placebo group may not fill the OS gap brought by osimertinib adjuvant treatment.

Third, early-stage EGFR-mutant lung cancers may benefit less from adjuvant EGFR-TKIs, reducing the overall population survival benefit. Based on the sensitivity analysis (Fig. 6B) and the pooled HR results of OS (Fig. 3), Li’s study, EVAN and ADAURA trials mostly contributed to this improvement in OS. The OS HR values from the three positive trials were 0.38, 0.32, and 0.49, respectively. Li’s study and the EVAN trial included only stage IIIA patients, and the ADAURA trial reported positive results only for stage II and IIIA patients when subgroup analysis for OS in overall population was conducted. In Li’s study [7], although the OS difference did not reach significance, the observed DFS benefit appeared to translate into a marginal OS benefit (41.6 vs. 32.6 months) with gefitinib in stage IIIA-N2 patients. The EVAN trial [15] showed that adjuvant erlotinib therapy significantly improved OS in resected stage IIIA patients compared with adjuvant chemotherapy (84.2 vs. 61.1 months). Indeed, the pooled OS data demonstrated that first-generation EGFR-TKI adjuvant therapy could prolong OS for stage III patients (Fig. 3D). In addition, osimertinib adjuvant therapy might be associated with significantly better OS for the EGFR-TKI-treated patients. As shown in Fig. 3C, when the ADAURA trial was included, an improvement in OS in stage II-III EGFR-mutant NSCLC patients was evident.

Several questions regarding EGFR-TKI adjuvant therapy remain to be solved. First, circulating tumor DNA (ctDNA) has been considered a potential predictor of minimal residual disease or disease recurrence and is used to identify molecular residual disease in localized lung cancer [30]. Further research is needed to investigate the value of ctDNA in defining molecular residual disease to direct the EGFR-TKI treatment duration, chemotherapy intervention, and provide a personalized adjuvant treatment plan. Second, it is unclear whether chemotherapy remains necessary in an adjuvant setting. That is, for patients with EGFR-mutant resected lung cancer, is the optimal adjuvant treatment modality EGFR-TKIs alone or a combination of EGFR-TKIs and chemotherapy? Most trials involving EGFR-TKI adjuvant therapy were designed to directly compare EGFR-TKIs with chemotherapy and aimed to change the standard adjuvant chemotherapy to adjuvant EGFR-TKI treatment alone without chemotherapy [10, 13–15]. Although the ADAURA trial demonstrated that osimertinib provided significant improvement in both DFS and OS regardless of whether patients did or did not receive prior adjuvant chemotherapy, compared with a placebo [11, 27], analysis regarding to whether adjuvant chemotherapy followed by osimertinib could further prolong survival compared with adjuvant osimertinib alone is lacking. Mechanistically, the EGFR-TKIs inhibit the growth of sensitive mutant tumor cells rather than eradicating potential micrometastases of EGFR wild-type cancer cells. Therefore, theoretically, combination therapy is more likely to eradicate highly heterogeneous tumor cells. However, whether prior adjuvant chemotherapy has impact on the sensitivity or efficacy of EGFR-TKI treatment is not clear. Although we extracted some data from patients who underwent adjuvant chemotherapy prior to EGFR-TKI treatment, it is still difficult to answer this question clearly due to the unavailable or limited of the raw data. Third, some future challenges for the ADAURA trial should be taken into consideration, such as acquired resistance mechanisms at relapse and subsequent treatment. Taken together, additional clinical trials are needed to address these questions.

The potential bias of this meta-analysis was that the inhomogeneous study design included patients with different treatment regimens, at different stages of disease, and with different treatment durations. To reduce the treatment regimen bias, we performed the present analysis grouped by two comparisons: adjuvant EGFR-TKIs versus placebo and adjuvant EGFR-TKIs versus chemotherapy. The different generations of EGFR-TKIs were also categorized and analyzed. Although we performed specific subgroup analyses of stage I, II, and III patients in terms of DFS and OS, the results were compromised by a lack of information on the stage of disease for each patient and the limited survival data. In addition, the median duration of EGFR-TKI treatment differed in each study, which could also cause some selection bias.

## Conclusion

To the best of our knowledge, this meta-analysis includes the largest sample size analyzed to date and determined the role of EGFR-TKIs in an adjuvant setting in patients with completely resected stage IB-IIIA EGFR-mutant NSCLC. Patients with resected EGFR-mutant NSCLC showed significantly improved DFS when treated with adjuvant EGFR-TKIs compared with non-EGFR-TKI treatment, regardless of EGFR mutation type or EGFR-TKI generation and stage, with tolerated AEs. The DFS advantage from first-generation EGFR-TKI adjuvant therapy can translate to an OS benefit in stage III NSCLC patients. Osimertinib may be a better candidate for adjuvant therapy than first-generation EGFR-TKIs, due to the lower recurrence rate and the potent OS benefit, even in early-stage patients. In addition, a 2-year treatment time with EGFR-TKI adjuvant therapy was found to be superior to ≤ 1-year adjuvant therapy; however, the optimal treatment duration of EGFR-TKIs at different stages of disease needs to be validated. Further studies are warranted to identify the optimal adjuvant treatment regimen, duration, and agent for patients with different clinical characteristics.

## Electronic supplementary material

Below is the link to the electronic supplementary material.

Supplementary Material 1

Supplementary Material 2

Supplementary Material 3

Supplementary Material 4

Supplementary Material 5

Supplementary Material 6

Supplementary Material 7

Supplementary Material 8

## Data Availability

The datasets used and/or analysed during the current study are available from the corresponding author on reasonable request.

## Abbreviations

AEs: Adverse events
ASCO: American Society of Clinical Oncology
CIs: Confidence intervals
DFS: Disease-free survival
EGFR: Epidermal growth factor receptor
EGFR-TKI: Epidermal growth factor receptor tyrosine kinase inhibitor
ESMO: European Society for Medical Oncology
HRs: Hazard ratios
NSCLC: Non-small-cell lung cancer
OS: Overall survival
RCTs: Randomized controlled trials
RR: Relative risk
