# Supplementary material for: Rational application of EGFR-TKI adjuvant therapy in patients with completely resected stage IB-IIIA EGFR-mutant NSCLC: a systematic review and meta-analysis of 11 randomized controlled trials
Source: BMC Cancer. 2023 Aug 1;23:719. doi: 10.1186/s12885-023-11194-6 (PMC10391763; doi:10.1186/s12885-023-11194-6)
Supplement: Supplementary file 2 — Supplementary Material 2 [file 12885_2023_11194_MOESM2_ESM.docx]

Table S2. Search strategy for PubMed, Embase, Cochrane Library and Chinese National Knowledge Infrastructure databases.

| Database | Search Strategy |
| --- | --- |
| PubMed | ((((Non-Small Cell Lung Cancer[MeSH Major Topic]) OR (((((((Non-Small Cell Lung Cancer[Title/Abstract]) OR (Non-Small Cell Lung Carcinoma[Title/Abstract])) OR (NSCLC[Title/Abstract])) OR (Non-Small Cell Lung Carcinoma[Title/Abstract])) OR (Non-Small-Cell Lung Carcinoma[Title/Abstract])) OR (Non-small Cell Lung Cancer[Title/Abstract])) OR (Non-Small-Cell Lung Carcinomas[Title/Abstract])) OR (Non-Small-Cell Lung Carcinoma[Title/Abstract]))))) AND ((((((tyrosine kinase inhibitor[Title/Abstract]) OR (TKI[Title/Abstract])) OR (epidermal growth factor receptor tyrosine kinases inhibitor)) [Title/Abstract] OR (EGFR-TKI[Title/Abstract])) OR ((erlotinib[Title/Abstract]) OR (gefitinib[Title/Abstract]) OR (icotinib[Title/Abstract]) OR (afatinib[Title/Abstract]) OR (dacomitinib[Title/Abstract]) OR (almonertinib[Title/Abstract]) OR (osimertinib[Title/Abstract]))) OR ((Adjuvant Drug Therapy[Title/Abstract]) OR (Adjuvant Therapy[Title/Abstract]) OR (Adjuvant treatment [Title/Abstract])) AND ((randomized controlled trial[Publication Type]) OR (controlled clinical trial[Publication Type]) OR (randomized[Title/Abstract]) OR (randomly [Title/Abstract])) |
| Embase | ('epidermal growth factor receptor-tyrosine kinase inhibitor':ti,ab OR 'egfr-tki':ti,ab OR 'egfr':ab,ti OR ('erlotinib':ab,ti OR 'gefitinib':ab,ti OR 'icotinib':ab,ti OR 'afatinib':ab,ti OR 'dacomitinib':ab,ti OR 'almonertinib':ab,ti OR 'osimertinib':ab,ti) OR 'adjuvant therapy':ab,ti OR 'adjuvant treatment':ab,ti) AND ('non small cell' AND ('lung'/exp OR lung) OR 'carcinoma, non small cell lung':ab,ti OR 'non-small-cell lung carcinoma':ab,ti OR 'lung carcinoma, non-small-cell':ab,ti OR 'non-small cell lung cancer':ab,ti) AND ('randomized controlled trial':mp OR 'controlled clinical trial':mp OR 'randomized':ab OR randomly':ab OR 'trial':ab) |
| Cochrane Library | ((MeSH descriptor: [Carcinoma, Non-Small-Cell Lung] explode all trees) OR (Non-Small Cell Lung Cancer):ti,ab,kw OR (NSCLC):ti,ab,kw OR (Non-Small Cell Lung Carcinoma):ti,ab,kw OR (Non Small Cell Lung Carcinoma):ti,ab,kw (Non-Small-Cell Lung Carcinoma):ti,ab,kw OR (Non-small Cell Lung Cancer):ti,ab,kw OR (Non-Small-Cell Lung Carcinomas):ti,ab,kw OR (Non-Small-Cell Lung Carcinoma):ti,ab,kw) AND ((epidermal growth factor receptor-tyrosine kinase inhibitor):ti,ab,kw OR (egfr-tki):ti,ab,kw OR (EGFR):ti,ab,kw OR (erlotinib):ti,ab,kw OR (gefitinib):ti,ab,kw OR (icotinib):ti,ab,kw OR (afatinib):ti,ab,kw OR (dacomitinib):ti,ab,kw OR (almonertinib):ti,ab,kw OR (osimertinib):ti,ab,kw OR (adjuvant therapy):ti,ab,kw OR (adjuvant treatment):ti,ab,kw) |
| Chinese National Knowledge Infrastructure databases | ((Subject: EGFR-TKI (precise)) OR (Subject: epidermal growth factor receptor tyrosine kinase inhibitors (precise)) OR (Subject: adjuvant therapy (fuzzy)) OR (Subject: Adjuvant Therapy (fuzzy)) OR (Subject: dacomitinib (precise)) OR (Subject: afatinib (accurate)) OR (Subject: icotinib (accurate)) OR (Subject: erlotinib (accurate)) OR (Subject: gefitinib (accurate)) OR (subject: almonertinib (accurate)) OR (subject: osimertinib (accurate))) AND ((Subject: lung cancer (precise)) OR (subject: lung tumors (fuzzy)) OR (Subject: chest tumors (fuzzy)) OR (Subject: Non-small cell lung cancer (precise))) AND (Published date: 2008-2023) |
