# Supplementary material for: Rational application of EGFR-TKI adjuvant therapy in patients with completely resected stage IB-IIIA EGFR-mutant NSCLC: a systematic review and meta-analysis of 11 randomized controlled trials
Source: BMC Cancer. 2023 Aug 1;23:719. doi: 10.1186/s12885-023-11194-6 (PMC10391763; doi:10.1186/s12885-023-11194-6)
Supplement: Supplementary file 3 — Supplementary Material 3 [file 12885_2023_11194_MOESM3_ESM.docx]

Table S2. Grade ≥3 AEs in the EGFR-TKI treatment group

| AEs | Number of trials | Events | Incidence (95% CI), % |
| --- | --- | --- | --- |
| Rash | 7 (7,8,10,13,14,15,18,19,20) | 35 | 4.3 (1.6-6.9) |
| Diarrhea | 8 (7,8,10,11,13,14,15,18,19) | 25 | 1.7 (0.8-2.7) |
| Stomatitis | 1 (11) | 6 | 1.8 (0.4-3.2) |
| Elevated ALT | 2 (13,14) | 28 | 11.9 (0-32.3) |
| Elevated AST | 2 (13,14) | 19 | 7.9 (0-20.6) |
| Pneumonia | 3 (11,13,15) | 6 | 1.2 (0.2-2.1) |
| Anorexia | 4 (7,8,11,14) | 5 | 0.7 (0.0-1.4) |
| Dry skin | 3 (10,11,19) | 3 | 0.4 (0.0-0.9) |
| Asthenia | 2 (8,11) | 2 | 0.4 (0.0-0.9) |
| Any | 8 (7,10,11,13,14,15,18,19,20) | 175 | 16.3 (10.0-22.7) |

Note: AEs, adverse events; EGFR-TKI, epidermal growth factor receptor tyrosine kinase inhibitor; ALT, alanine transaminase; AST, aspartate transaminase.
