# Supplementary material for: Rational application of EGFR-TKI adjuvant therapy in patients with completely resected stage IB-IIIA EGFR-mutant NSCLC: a systematic review and meta-analysis of 11 randomized controlled trials
Source: BMC Cancer. 2023 Aug 1;23:719. doi: 10.1186/s12885-023-11194-6 (PMC10391763; doi:10.1186/s12885-023-11194-6)
Supplement: Supplementary file 4 — Supplementary Material 4 [file 12885_2023_11194_MOESM4_ESM.docx]

**Figure legends**

Fig. S1. Effects of adjuvant first- and third-generation EGFR-TKI on DFS. Forest plot of the HRs of DFS for adjuvant TKI vs non-TKI therapy in resected patients with EGFR-mutant NSCLC (A); Forest plot of the RRs of the 2- and 3-year DFS rates for adjuvant TKI vs non-TKI therapy in these patients (B, C). EGFR-TKI, epidermal growth factor receptor tyrosine kinase inhibitor; DFS, disease-free survival; HR, hazard ratio; RR, relative risk; NSCLC, non-small-cell lung cancer.

Fig. S2. Forest plot of the HRs of DFS for adjuvant TKI vs non-TKI therapy for resected patients with stage II-III disease (A) and EGFR exon 19 deletion and L858R mutations (B). DFS, disease-free survival; HR, hazard ratio; RR, relative risk; NSCLC, non-small-cell lung cancer.

Fig. S3. Effects of adjuvant EGFR-TKI on OS. Forest plot of the HRs of OS for adjuvant TKI vs non-TKI therapy for resected patients with IB-III stage EGFR-mutant NSCLC (A) and for all stage II-III patients with different generations of EGFR-TKI treatment (C); Forest plot of the RRs for the 5-year OS rates (B) between different groups. EGFR-TKI, epidermal growth factor receptor tyrosine kinase inhibitor; OS, overall survival; HR, hazard ratio; RR, relative risk; NSCLC, non-small-cell lung cancer.

Fig. S4. Risk of bias for each included study based on the evaluation elements listed in the Cochrane Risk of Bias 2 (RoB2) tool: risk of bias summary (A), risk of bias graph (B).
