# Supplementary figures and images for: Rational application of EGFR-TKI adjuvant therapy in patients with completely resected stage IB-IIIA EGFR-mutant NSCLC: a systematic review and meta-analysis of 11 randomized controlled trials
Source: BMC Cancer. 2023 Aug 1;23:719. doi: 10.1186/s12885-023-11194-6 (PMC10391763; doi:10.1186/s12885-023-11194-6)

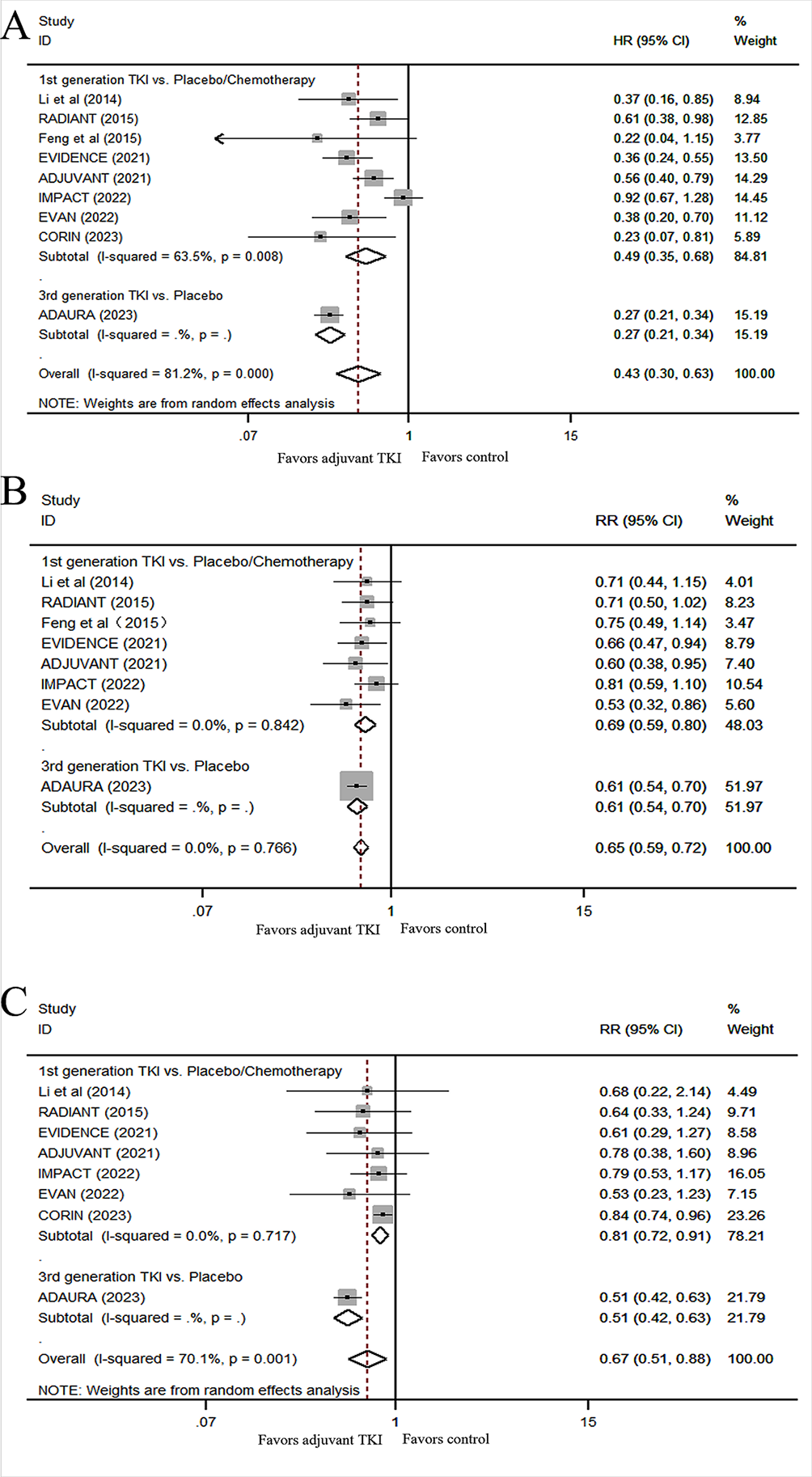

Supplement: Supplementary file 5 — Supplementary Material 5 [file 12885_2023_11194_MOESM5_ESM.tif]

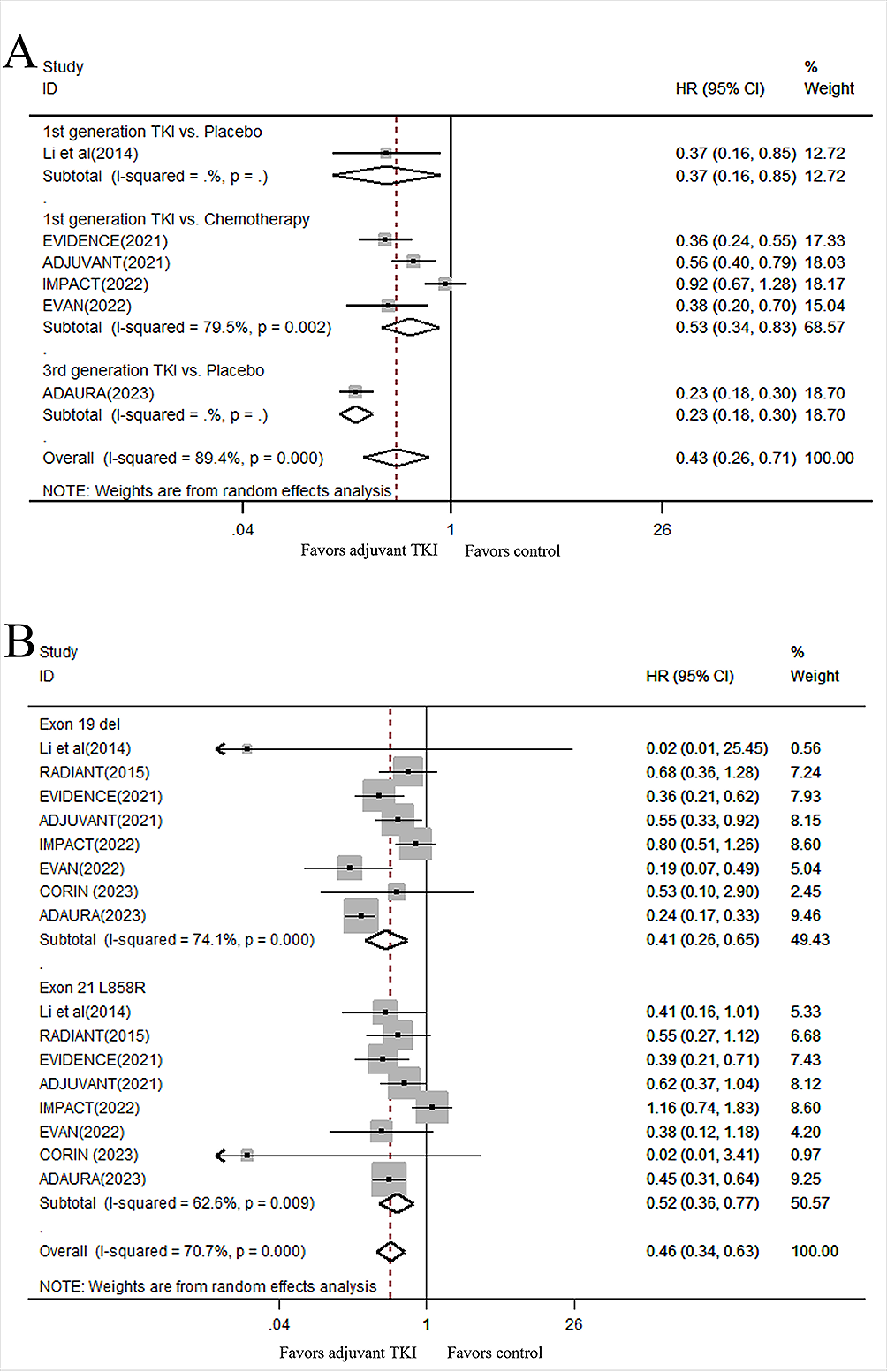

Supplement: Supplementary file 6 — Supplementary Material 6 [file 12885_2023_11194_MOESM6_ESM.tif]

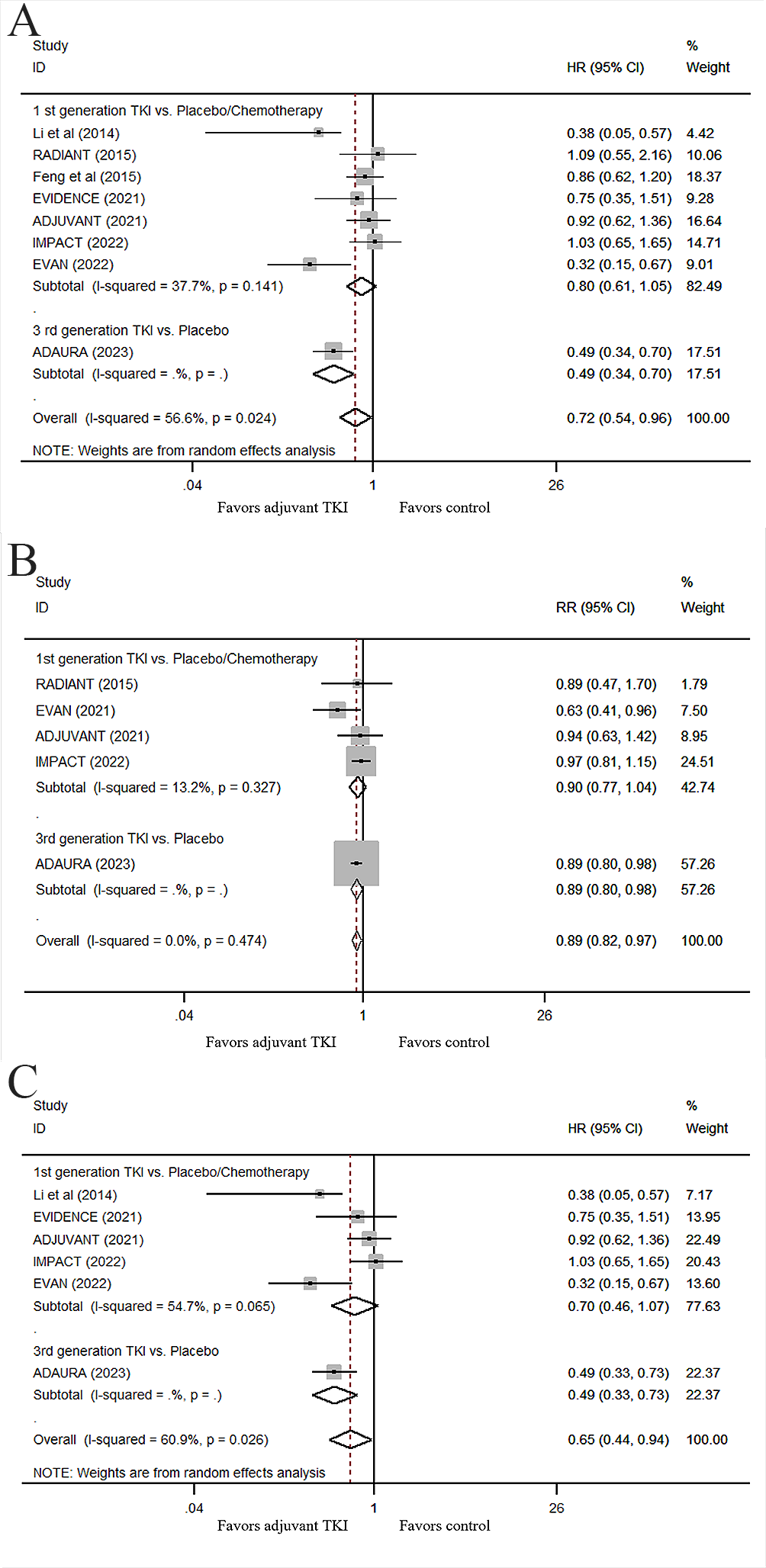

Supplement: Supplementary file 7 — Supplementary Material 7 [file 12885_2023_11194_MOESM7_ESM.tif]

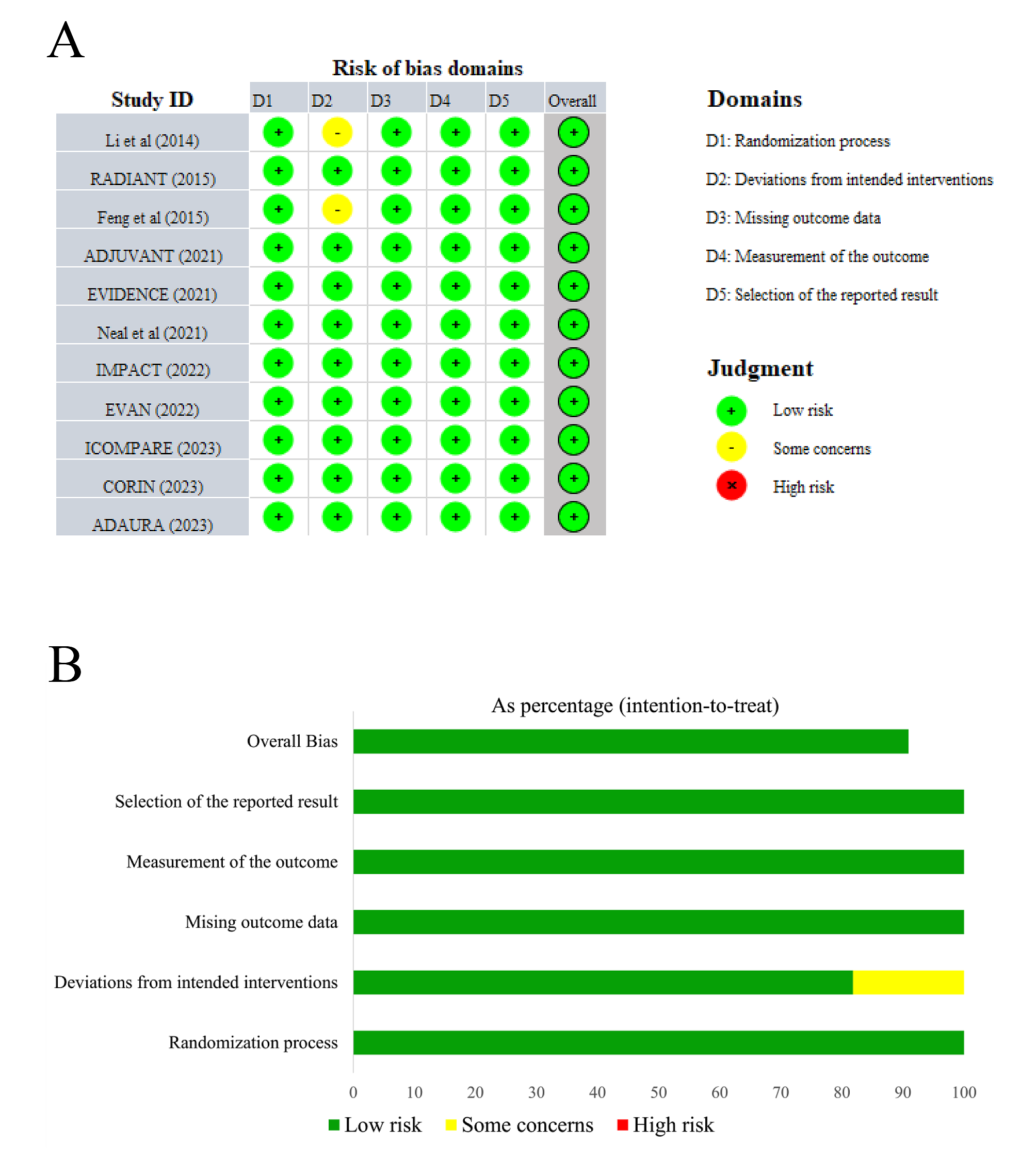

Supplement: Supplementary file 8 — Supplementary Material 8 [file 12885_2023_11194_MOESM8_ESM.tif]
